# Prostate Surface Distension and Tumor Texture Descriptors From Pre-Treatment MRI Are Associated With Biochemical Recurrence Following Radical Prostatectomy: Preliminary Findings

**DOI:** 10.3389/fonc.2022.841801

**Published:** 2022-05-20

**Authors:** Rakesh Shiradkar, Soumya Ghose, Amr Mahran, Lin Li, Isaac Hubbard, Pingfu Fu, Sree Harsha Tirumani, Lee Ponsky, Andrei Purysko, Anant Madabhushi

**Affiliations:** ^1^Department of Biomedical Engineering, Case Western Reserve University, Cleveland, OH, United States; ^2^GE Global Research, Niskayuna, NY, United States; ^3^Department of Urology, University Hospitals Cleveland Medical Center, Cleveland, OH, United States; ^4^Department of Population and Quantitative Health Sciences, Case Western Reserve University, Cleveland, OH, United States; ^5^Department of Radiology, University Hospitals Cleveland Medical Center, Cleveland, OH, United States; ^6^Department of Abdominal Imaging and Nuclear Radiology, Imaging Institute, Cleveland Clinic, Cleveland, OH, United States

**Keywords:** magnetic resonance imaging, prostate cancer, retrospective studies, prostatectomy, artificial intelligence, machine learning

## Abstract

**Objective:**

To derive and evaluate the association of prostate shape distension descriptors from T2-weighted MRI (T2WI) with prostate cancer (PCa) biochemical recurrence (BCR) post-radical prostatectomy (RP) independently and in conjunction with texture radiomics of PCa.

**Methods:**

This retrospective study comprised 133 PCa patients from two institutions who underwent 3T-MRI prior to RP and were followed up with PSA measurements for ≥3 years. A 3D shape atlas-based approach was adopted to derive prostate shape distension descriptors from T2WI, and these descriptors were used to train a random forest classifier (*C_S_*) to predict BCR. Texture radiomics was derived within PCa regions of interest from T2WI and ADC maps, and another machine learning classifier (*C_R_*) was trained for BCR. An integrated classifier *C_S_*_+_*_R_* was then trained using predictions from *C_S_* and *C_R_*. These models were trained on D_1_ (*N* = 71, 27 BCR+) and evaluated on independent hold-out set D_2_ (*N* = 62, 12 BCR+). *C_S_*_+_*_R_* was compared against pre-RP, post-RP clinical variables, and extant nomograms for BCR-free survival (bFS) at 3 years.

**Results:**

*C_S_*_+_*_R_* resulted in a higher AUC (0.75) compared to *C_R_* (0.70, *p* = 0.04) and *C_S_* (0.69, *p* = 0.01) on D_2_ in predicting BCR. On univariable analysis, *C_S_*_+_*_R_* achieved a higher hazard ratio (2.89, 95% CI 0.35–12.81, *p* < 0.01) compared to other pre-RP clinical variables for bFS. *C_S_*_+_*_R_*, pathologic Gleason grade, extraprostatic extension, and positive surgical margins were associated with bFS (*p* < 0.05). *C_S_*_+_*_R_* resulted in a higher C-index (0.76 ± 0.06) compared to CAPRA (0.69 ± 0.09, *p* < 0.01) and Decipher risk (0.59 ± 0.06, *p* < 0.01); however, it was comparable to post-RP CAPRA-S (0.75 ± 0.02, *p* = 0.07).

**Conclusions:**

Radiomic shape descriptors quantifying prostate surface distension complement texture radiomics of prostate cancer on MRI and result in an improved association with biochemical recurrence post-radical prostatectomy.

## 1 Introduction

An estimated 30%–35% of prostate cancer (PCa) patients experience biochemical recurrence (BCR) within 10 years post-radical prostatectomy (RP) (1). The occurrence of BCR is often found to be associated with metastasis (2) and PCa-specific mortality (3). Several predictors of BCR have been presented including pre-treatment CAPRA (4), post-surgical CAPRA-S (5), and Decipher risk (6). However, these models use invasive or post-treatment factors, are site dependent, and do not exclusively capture tumor heterogeneity and morphology. Non-invasive pre-treatment image-based prediction of BCR-free survival (bFS) may allow for treatment intensification or closer surveillance (7).

Multiparametric magnetic resonance imaging (mpMRI) is now increasingly used for PCa detection, staging, and prediction of the risk of BCR (8–17). Radiomic texture features provide an alternative representation for characterizing tumor heterogeneity and have been shown to improve PCa risk characterization (18–21) and also prognosticate BCR (22–24). However, they are susceptible to scanner variations, acquisition protocols, image artifacts, and non-standardized image intensities (25, 26).

There is evidence to suggest that cancerous lesions tend to induce mechanical stress in the surrounding tissue (27, 28). In the prostate, stresses induced by the tumors impact neighboring benign tissue (29) and cause deformation which may in turn impact the shape of the prostate capsule. Previous studies (30, 31) explored the idea of quantifying shape distension of the prostate between more and less aggressive diseases. Rusu et al. (30) have shown that prostate shape on T2-weighted MRI (T2WI) was significantly different between patients with and without cancerous lesions. Ghose et al. (31, 32) have shown that statistically significant differences in the shape of the prostate were observed between BCR+ and BCR− patients on T2WI. This now leads us to the hypothesis that radiomic descriptors that quantify differential distension of prostate shape may be associated with BCR outcome post-RP.

In this work, we present a new approach to quantify prostate distension in terms of radiomic shape descriptors from pre-treatment T2WI using a 3D shape atlas-based method. We seek to evaluate the association of these shape descriptors with BCR post-RP at 3 years. Since radiomic texture features of PCa from pre-treatment T2WI and apparent diffusion coefficient (ADC) maps have already been shown to be associated with BCR (17, 22–24), we evaluate the combination of prostate shape and tumor texture radiomics for their association with bFS. Additionally, we also sought to evaluate whether radiomic shape descriptors, which are less dependent on MRI intensities, are possibly more robust and resilient to scanner- and site-specific variations that could more substantially impact texture-based descriptors (25, 26). This was done by evaluating our approach using data from multiple cohorts acquired from two different sites. We also compared our integrated shape and texture radiomics approach against routine clinical variables and extant nomograms for predicting bFS.

## 2 Materials and Methods

### 2.1 Patient Selection and Data Characteristics

**Figure 1:**
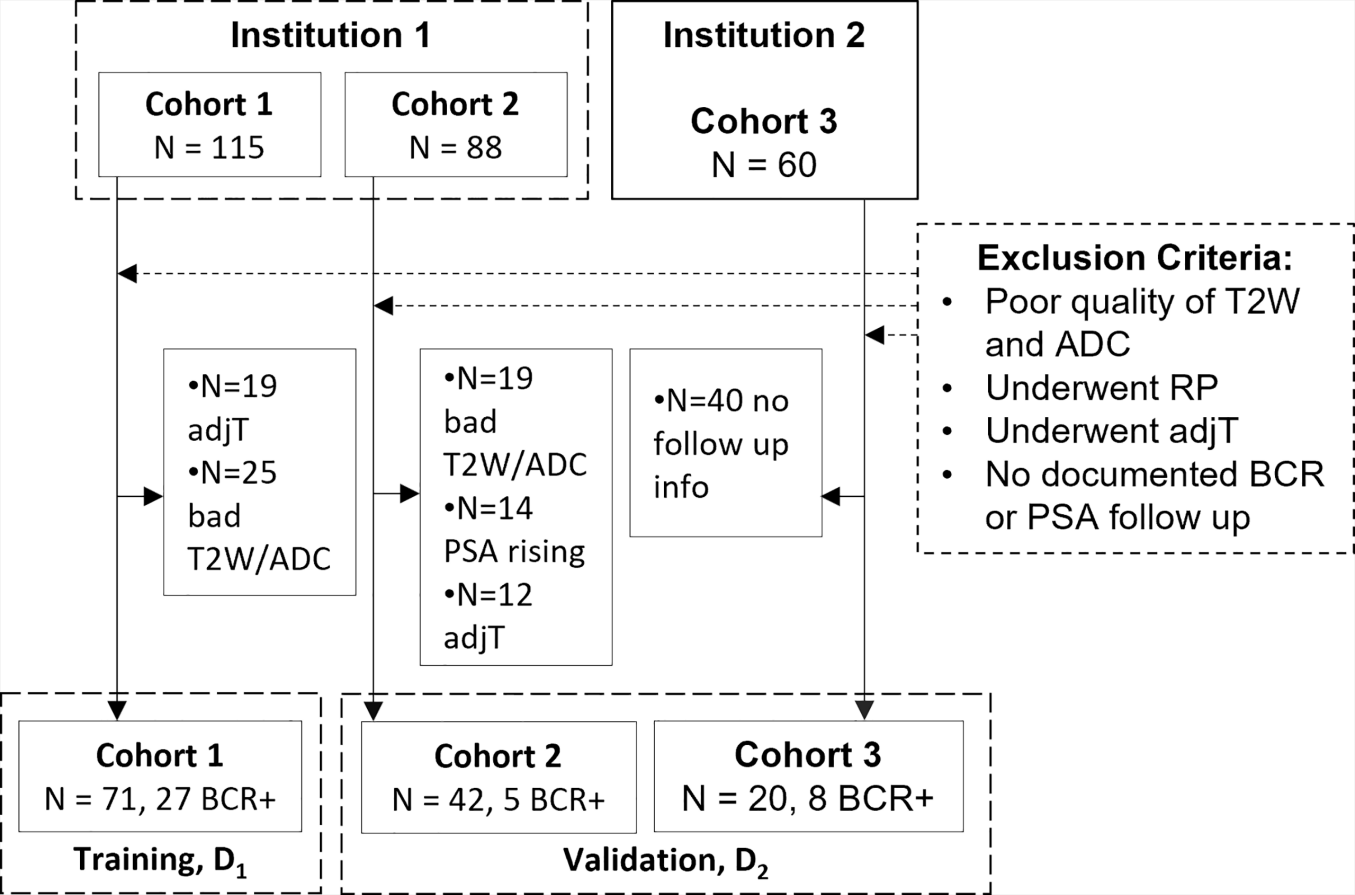
Flowchart of patient selection from two different institutions. adjT, adjuvant or neoadjuvant therapy; RP, radical prostatectomy; BCR, biochemical recurrence; PSA, prostate-specific antigen.

This retrospective study included patients from two different institutions (I_1_ and I_2_), compliant with HIPAA regulations and approved by the local IRB, with a waiver of informed consent. A total of 263 patients from three cohorts (C_1_–C_3_) were identified with biopsy-confirmed PCa who underwent 3T mpMRI prior to RP. Patient records between 2009 and 2017 were reviewed and included in the study if they 1) underwent RP without additional therapies, 2) followed up for at least 3 years and had documented BCR (BCR+) defined as two consecutive readings of prostate-specific antigen (PSA) ≥0.2 ng/ml post-RP [18] or no BCR (BCR−), and 3) had axial T2WI and ADC maps without acquisition artifacts. *N* = 133 studies identified through this process were partitioned into a training set D_1_ and independent hold-out validation set D_2_ as illustrated in **Figure 1**. After RP, all patients underwent periodic follow-up according to established clinical protocol (3–6 months in the first year and 6–12 months in the following years). BCR− patients were censored at their last follow-up date for survival analysis.

Biparametric MRI protocol was used in this study that included T2WI (in-plane: 0.3 mm, slice thickness: 3 mm, TR/TE: 3,802–5,151/105–115 ms) and DWI (1.4 mm, 3 mm, 3,751–4,880/50–74 ms, *b*-values: 0–2,000 s/mm^2^) along with ADC maps acquired *via* an endo-rectal coil (Achieva, Philips, Best, Netherlands) in C_1_, T2WI (0.5 mm, 3 mm, 3,730/121 ms) and DWI (1.6 mm, 3 mm, 4,700/86 ms, 0–1,500 s/mm^2^) acquired *via* a pelvic-phased array coil (PPAC) (Skyra, Siemens, Erlangen, Germany) in C_2_, and T2WI (0.6 mm, 3 mm, 7,200/96 ms) and DWI (1.2 mm, 3 mm, 7,900/88 ms, 0–1,400 s/mm^2^) *via* PPAC (Skyra, Siemens, Erlangen, Germany). Additional sequences, such as T1-weighted, dynamic contrast-enhanced (DCE), were acquired but not analyzed in the current study. Detailed dataset characteristics are provided in **Table 1** and imaging parameters in **Table 2**.

**Table 1:** Summary of the patient characteristics in different cohorts in terms of clinical variables.

Parameter	Training D_1_	Validation D_2_	
	Cohort 1	Cohort 2	Cohort 3
*N* (patients)	71	42	20
*N* (BCR+)	27	5	8
*N* (BCR−)	44	37	12
Median age (range), years	59 (47–79)	64 (42–76)	61 (47–86)
Mean PSA (range), ng/ml	10 (1–58)	10.6 (1.8–88.3)	9 (1.2–69.4)
Lesion volume (cm^3^)	3.93 ± 6.47	3.34 ± 5.13	2.20 ± 5.05
Prostate volume (cm^3^)	35.65 ± 12.37	38.81 ± 19.04	40.83 ± 16.99
Follow-up (months)	43 ± 28	30 ± 24	33 ± 18
biopsy Gleason Grade Group and RP Gleason Grade Group (*N*)			
1	15	7	3
2	20	27	6
3	8	13	5
4	10	3	4
5	18	6	2
RP pGG			
1	6	4	3
2	22	24	8
3	13	15	4
4	7	3	2
5	18	10	3
PI-RADS-v2.0			
1	0	1	0
2	10	8	2
3	6	3	2
4	21	10	6
5	34	34	10
EPE			
Yes	33	21	8
No	30	21	12
N/A	8	0	0
SVI			
Yes	24	5	4
No	42	37	16
N/A	5	0	0
PSM			
Yes	0	20	6
No	0	22	14
N/A	71	0	0
LNI			
Yes	12	15	1
No	41	22	18
N/A	18	5	1
Decipher risk			
Low	N/A	20	N/A
Intermediate	N/A	5	N/A
High	N/A	17	N/A

BCR, biochemical recurrence; RP, radical prostatectomy ;PI-RADS, Prostate Imaging Reporting and Data System; EPE, Extra Prostatic Extension; SVI, Seminal Vesicle Invasion; PSM, Positive Surgical Margin; LNI , Lymph Node Invasion; N/A, Not Available.

**Table 2:** Imaging parameters of scans used in this study.

Parameter	Institution 1		Institution 2
	Scanner 1	Scanner 2	
Manufacturer	Philips Medical Systems, Best, Netherlands	Siemens Healthcare, Erlangen, Germany	Siemens Healthcare, Erlangen, Germany
Model	3T Achieva	3T Skyra	3T Skyra
Coils	ERC	PPAC	PPAC
T2-weighted sequence (T2WI)			
TR/TE, ms	3,802–5,151/105–115	3,730/121	7,200/96
Resolution, mm^3^	0.3 × 0.3 × 3	0.5 × 0.5 × 3	0.6 × 0.6 × 3
Diffusion-weighted imaging (DWI)			
TR/TE, ms	3,751–4,880/50–74	4,700/86	7,900/88
Resolution, mm^3^	1.4 × 1.4 × 3	1.6 × 1.6 × 3	1.2 × 1.2 × 3
*b*-values	0, 500, 1,000, 1,500, 2,000	0, 400, 900, 1,500	50, 600, 1,000, 1,400

TR, reconstruction time; TE, echo time.

### 2.2 Image Pre-Processing and Tumor Segmentation

A board-certified radiologist (10 years of experience in prostate imaging) reviewed the MRI scans in D_1_. They used the 3D Slicer software (33) to delineate prostate capsule and dominant PCa lesions on T2WI using histopathology template reports from RP as reference. Prostate and lesion delineation in D_2_ was performed by two board-certified radiologists (8 and 10 years of experience in prostate imaging) in a similar manner and assigned PI-RADS-v2.1 scores (34).

### 2.3 Methodology

**Figure 2:**
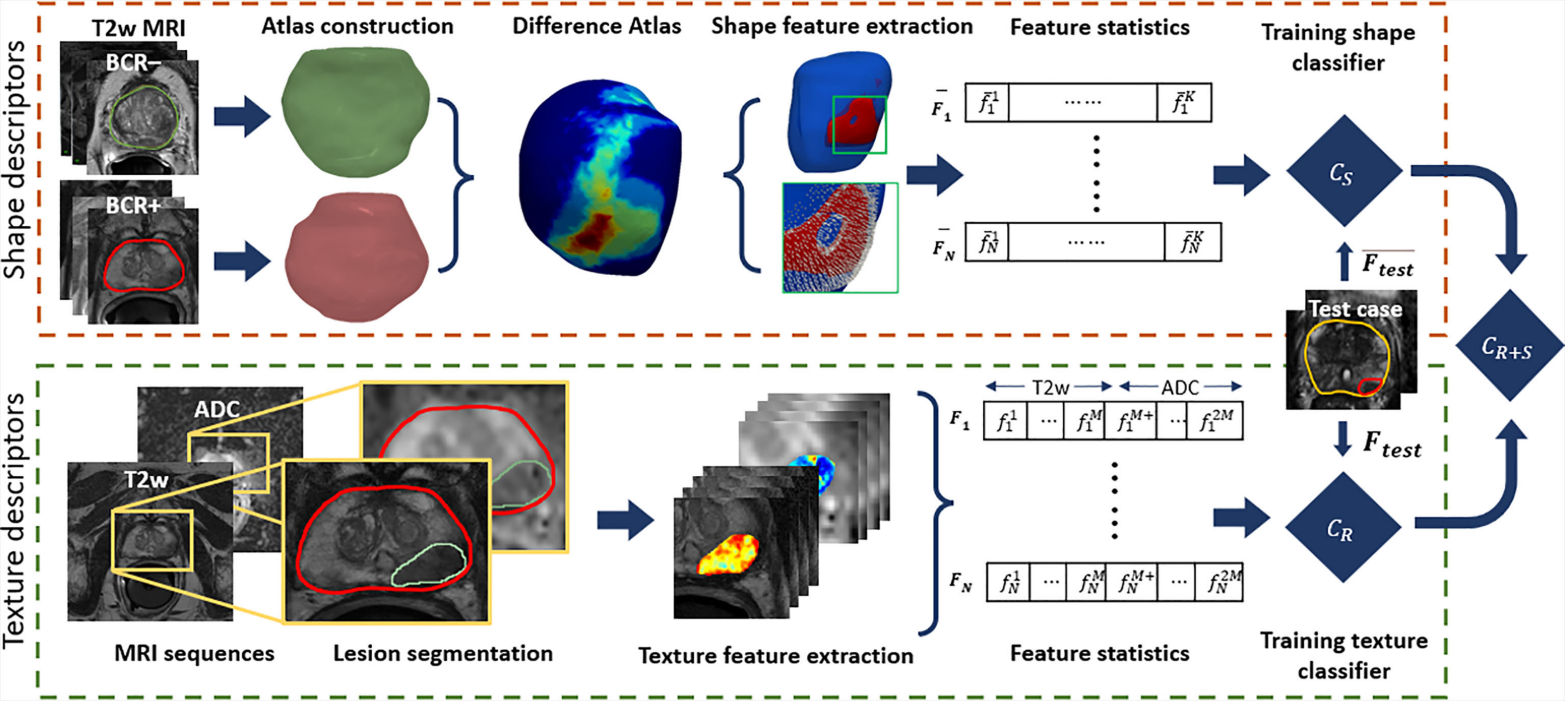
Illustration of the radiomic shape and lesion texture descriptors pipeline used in this study. The shape descriptors are computed from a surface of interest determined from a 3D differential shape atlas between BCR+ and BCR− cohorts on T2WI. These are used to train a model for predicting BCR-free survival and integrated with another model trained using lesion texture descriptors derived from T2WI and ADC maps.

The pipeline for radiomic prostate shape and lesion texture, along with their combination to evaluate the association with BCR, is illustrated in **Figure 2**. The term surface of interest (SOI) is used in the context of shape descriptors to describe a region on the 3D surface, while the region of interest (ROI) is used in the context of PCa lesions on 2D MRI slices.

#### 2.3.1 Computation of a Consensus Surface of Interest

Prostate segmentations on T2WI within D_1_ were co-registered via rigid registration followed by a deformable registration to create a 3D shape atlas for the BCR+ and BCR− cohorts. A spatially contextual SOI of the prostate capsule was uniquely identified from statistically significant shape differences between BCR+ and BCR− atlases as described earlier by Ghose et al. (31) (details in the **Supplementary Material**). To minimize the effect of the choice of template in establishing the SOI, the entire process was repeated *N* times, each time with a different set of BCR+ and BCR− atlases generating an SOI*_i_* (where *I* = 1, 2, 3… *N*). Here, *N* was chosen as 27, equal to the number of patients in BCR+ class of D_1_. The individual SOI*_i_* was again co-registered to a common frame of reference using a rigid transformation. Next, a consensus SOI (SOI_C_) was computed as a mean of all the SOI*_i_* and the mean volume was binarized at a threshold of 0.5.

#### 2.3.2 Prostate Shape Distension Features

**Figure 3:**
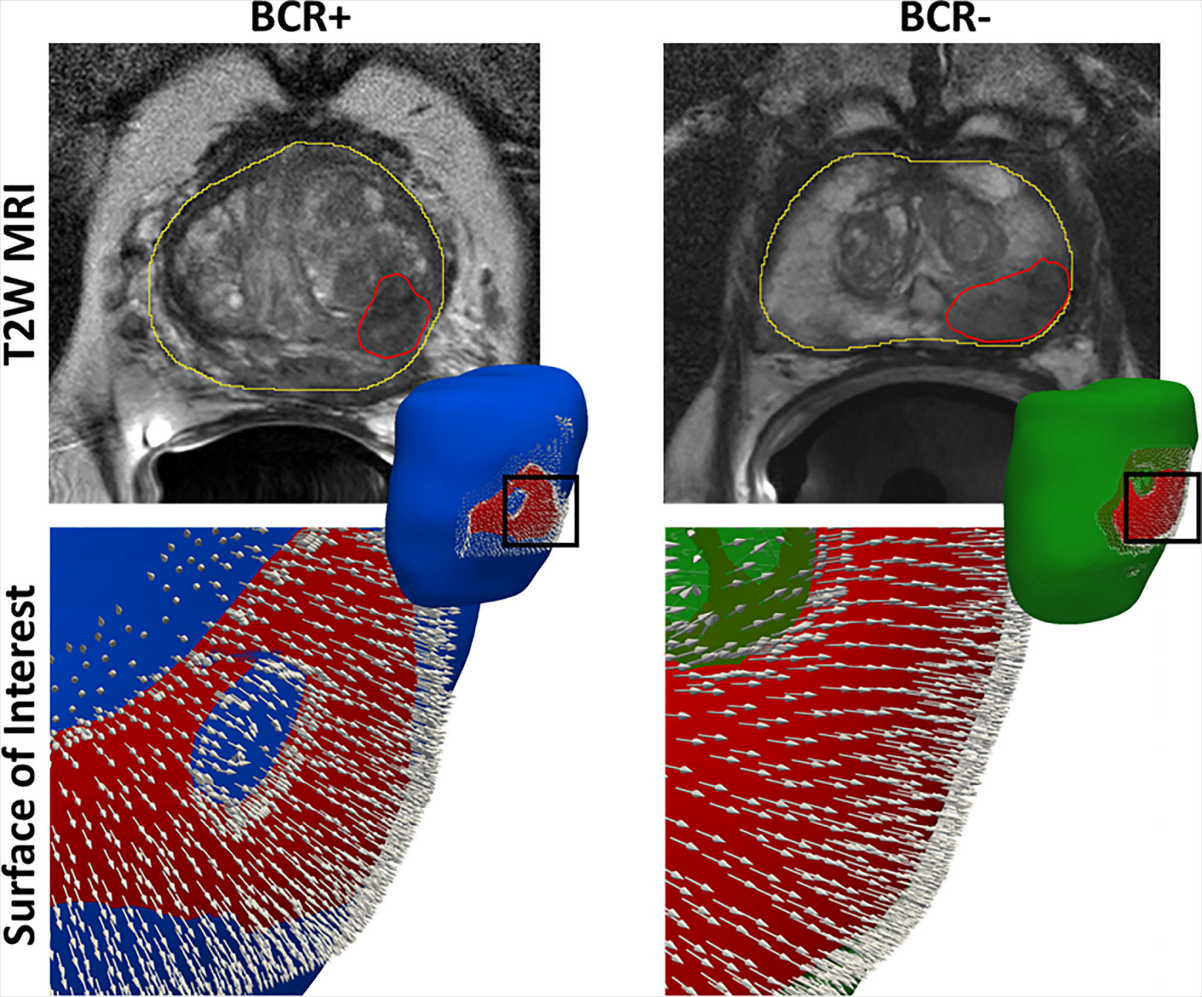
The surface of interest (SOI_C_) in red overlaid onto the prostate mesh of a BCR+ patient and a BCR− patient. We observe stronger surface distension in terms of more dense and divergent normal features in the BCR+ patient compared to the BCR− patient.

Differential distension of the prostate capsule for each patient is quantified using the magnitudes of Gaussian curvature (*κ*) of the surface and orientation of the surface normal (*θ*, *ϕ*) at vertices of the prostate mesh. The Gaussian curvature measures the intrinsic degree of curvedness of a surface, and positive values indicate a greater differential expansion at the center, while negative values indicate expansion at the edges (35). Surface normal quantifies the local orientation of the surface. These features (*κ*, *θ*, and *ϕ*) are meaningful when extracted within the SOI_C_ that comprises those vertices where statistically significant shape differences between BCR+ and BCR− prostates were observed. Further, the SOI_C_ was cropped to include the mid-gland region alone after mapping it to individual prostate meshes to minimize the effect of inter-reader variations in prostate segmentation at the apex and base. The SOI_C_ overlaid onto a BCR+ and a BCR− prostate is illustrated in **Figure 3**. Surface normal orientation (*θ*, *ϕ*) provides direction of the prostate distension and surface mean curvature (*κ*) quantifies local shape deformation, and these were extracted for every vertex on the mapped SOI_C_. For each patient, at each vertex, a vector of *κ*, *θ*, and *ϕ* was derived and a set of four statistical measures, namely, mean, standard deviation, skewness, and kurtosis, were computed resulting in 4 × 3 = 12 radiomic shape descriptors per patient.

#### 2.3.3 Radiomic Texture Features of Prostate Cancer

A set of 75 radiomic features were extracted on a per-voxel basis from each of the standardized T2WI and ADC maps within the radiologist-delineated PCa ROIs. These include first- and second-order statistics, Gabor (36), Laws (37), Haralick (38), and CoLlAGe (39) features. These features characterize the underlying tissue heterogeneity and have previously been shown to be prognostic of BCR (17, 22–24). Four statistics, namely, mean, standard deviation, skewness, and kurtosis, were calculated for voxel-wise radiomic feature within each PCa ROI resulting in 75 × 2 × 4 = 600 radiomic texture descriptors per patient. Strongly correlated features and unstable features were eliminated, and mRMR feature selection (40) was employed to identify a subset of features associated with BCR (details in the **Supplementary Material**).

### 2.4 Statistical Analysis and Comparison

A random forest machine learning classifier (*C_S_*) with 50 trees and 5 as the maximum tree depth was trained using prostate distension features from D_1_ to predict the binary outcome of BCR. The random forest classifier was chosen to ensure that parametric assumptions are not made for the distribution of shape distension features. A texture radiomics classifier (*C_R_*) was trained in a similar manner as *C_S_*.

A linear regression model (*C_S_*_+_*_R_*) was trained on predictions from *C_S_* and *C_R_* using patients from D_1_. The trained models (*C_S_*, *C_R_*, *C_S_*_+_*_R_*) were evaluated using patients from the independent validation test set D_2_. Kruskal–Wallis test was employed to assess differences in radiomic shape and texture features between the cohorts without a parametric assumption. Youden’s index was used to determine the optimal operating point within receiver-operating characteristics (ROC) analysis to convert the estimated posterior class probabilities to dichotomous labels (BCR+ and BCR−). DeLong’s test was used to compare the AUCs between the classification models.

Univariable and multivariable analyses of *C_S_*_+_*_R_* were conducted along with pre-RP clinical parameters including age, PI-RADS-v2, PSA, and biopsy Gleason grade (bGG) on D_1_. Post-RP pathological Gleason grade (pGG), extraprostatic extension (EPE), seminal vesical invasion (SVI), and positive surgical margins (PSM) were compared on patients from D_2_. A penalized Cox proportional hazards (CPH) regression model was used for this purpose given the class imbalance in D_2_. The model *C_S_*_+_*_R_* was also compared with other nomograms including CAPRA and CAPRA-S and the genomic assay Decipher on subsets of patients for whom sufficient clinical variables were available to estimate BCR-free survival on D_2_. Wald test was used to determine statistical significance with *p-*values under 0.05, and the concordance index (C-index) and hazard ratios (HRs) were computed. Kaplan–Meier survival curves were estimated to determine the differences in BCR-free survival based on predictions of *C_S_*_+_*_R_* and comparative nomograms. The log-rank test was used to determine statistical significance (*p* < 0.05).

## 3 Results

### 3.1 Evaluation of Shape and Texture Radiomics for Their Association With BCR

**Figure 4:**
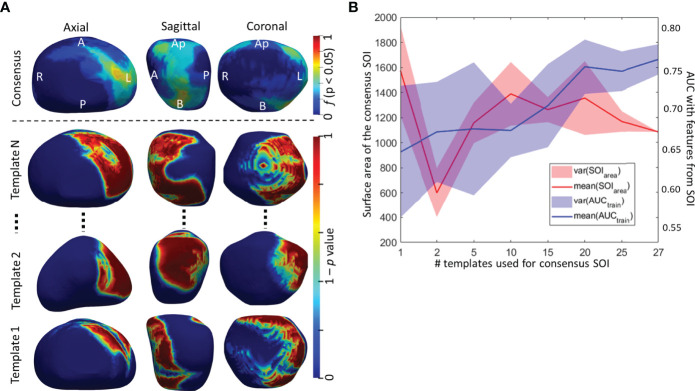
**(A)** Surface of interest (SOI) determined from individual templates and a consensus SOI_C_ derived by averaging individual SOIs. **(B)** The surface area and AUC from the predictive model trained radiomic shape descriptors as a function of the number of SOIs used in building the consensus SOI_C_.

The top radiomic shape and tumor texture descriptors based on their random forest Gini feature importance in the training set D_1_ are listed in **Table 3**. Shape descriptors were consistent between the sites (*p* > 0.05), whereas all radiomic texture features except Haralick had significant variations (*p* < 0.05). Shape descriptors from the entire prostate mesh resulted in a lower AUC of 0.58 ± 0.08, *p* < 0.01, compared to using those from the SOI_C_. Also, shape features from the SOI_C_ within the mid-gland region improved AUCs compared to those from the entire SOI_C_ (AUC = 0.75 vs. 0.78, *p* = 0.04), and the mid-gland SOI_C_ was used in all subsequent experiments. *C_S_* trained using individual SOIs resulted in AUC = 0.64 ± 0.08 on D_1_ in distinguishing BCR+ and BCR−, while the consensus SOI_C_ resulted in improved performance (AUC = 0.78, *p* < 0.01) (**Figure 4**). On the hold-out validation set D_2_, the AUC using models trained from individual SOIs was 0.67 ± 0.12, while that from the SOI_C_ was 0.69 (*p* = 0.02). Inter-reader variations in shape descriptors were evaluated on D_2_ and no significant differences were observed (*p* > 0.05) (**Supplementary Figure 2**).

**Table 3:** Variations in the top 5-ranked radiomic shape and texture descriptors according to Gini importance between the cohorts.

	Feature	Gini importance (cohort 1)	Statistics across cohorts			
			Cohort 1 (mean ± std)	Cohort 2 (mean ± std)	Cohort 3 (mean ± std)	*p*-value (Kruskal–Wallis)
Radiomic shape	Normal_th_kt	1.26	0.68 ± 0.21	0.67 ± 0.16	0.64 ± 0.13	**0.25**
	Normal_phi_sk	1.14	0.52 ± 0.14	0.54 ± 0.09	0.52 ± 0.13	**0.37**
	Normal_phi_kt	0.83	0.63 ± 0.18	0.64 ± 0.12	0.66 ± 0.12	**0.23**
	Curvature_mn	0.87	0.26 ± 0.13	0.26 ± 0.13	0.29 ± 0.17	**0.72**
	Curvature_kt	0.81	0.27 ± 0.12	0.25 ± 0.12	0.28 ± 0.16	**0.7**
Radiomic texture	Haralick_IM_T2	1.41	0.34 ± 0.18	0.31 ± 0.14	0.34 ± 0.18	**0.99**
	Laws_edge_T2	1.07	0.44 ± 0.24	0.43 ± 0.19	0.56 ± 0.15	<0.01
	CoLlAGe_ent_ADC	0.9	0.71 ± 0.16	0.54 ± 0.24	0.71 ± 0.16	0.03
	Haralick_en_ADC	0.88	0.26 ± 0.14	0.51 ± 0.19	0.75 ± 0.13	<0.01
	Gabor_T2	0.84	0.69 ± 0.15	0.42 ± 0.21	0.71 ± 0.12	<0.01

The values in bold indicate no statistical significance *p* < 0.05. This implies that the feature descriptors were less affected by inter-site variations.

The BCR prediction model (*C_R_*) using radiomic texture descriptors of the PCa lesion resulted in a cross-validation AUC = 0.76 ± 0.09 on D_1_ and AUC = 0.70 on D_2_. The integrated model (*C_R+S_*) resulted in a significantly higher (*p* < 0.05) AUC both on D_1_ (0.85 ± 0.08) and D_2_ (0.75) compared to both *C_S_* and *C_R_*, respectively. The AUC, sensitivity, and specificity of the models *C_S_*, *C_R_*, and *C_R+S_* for predicting BCR status are summarized in **Table 4**.

**Table 4:** Classification performance of prostate distension, lesion texture, and integrated classifiers for predicting biochemical recurrence-free survival.

Classifier	D_1_			D_2_		
	AUC (std)	Sensitivity	Specificity	AUC (std)	Sensitivity	Specificity
*C_Si_* (templates)	0.64 (0.58–0.72)	0.63 (0.56–0.79)	0.71 (0.56–0.88)	0.67 (0.52–0.71)	0.49 (0.44–0.65)	0.61 (0.49–0.73)
*C_S_* (consensus)	0.78 (0.69–0.82)	0.67 (0.59–0.71)	0.75 (0.69–0.88)	0.69	0.59	0.65
*C_R_*	0.76 (0.73–0.88)	0.72 (0.63–0.78)	0.86 (0.73–0.88)	0.7	0.6	0.66
*C_S_*_+_*_R_*	0.85 (0.76–0.93)	0.65 (0.61–0.75)	0.82 (0.77–0.91)	0.75	0.65	0.58

### 3.2 Comparison With Clinical Parameters and Extant Nomograms for Predicting bFS at 3 Years

On univariable analysis for predicting bFS, *C_S_*_+_*_R_* predictions resulted in the highest HR of 2.91 (95% CI 1.45–11.51, *p* = 0.02) on D_1_ compared to other pre-treatment clinical variables including age, PSA, bGG, and PI-RADS-v2. On multivariable analysis, *C_S_*_+_*_R_* and bGG showed independent predictive value (*p* < 0.05) for bFS (**Table 5**). Post-RP Gleason grade (pGG), EPE, and PSM had higher HRs of 2.63 (95% CI 1.16–5.93, *p* = 0.01), 2.51 (95% CI 1.06–11.26, *p* = 0.04), and 2.86 (95% CI 1.32–30.26, *p* = 0.03) on D_2_. The C-index followed the same trend with *C_S_*_+_*_R_* achieving the highest (0.76, *p* = 0.03) among pre-treatment clinical variables; however, it was lower than pGG (0.82, *p* = 0.01) (**Table 5**).

**Table 5:** Univariable and multivariable analyses for predicting BCR-free survival with pre-surgical variables (*N* = 71, *N*_BCR+_ = 27, *N*_BCR−_ = 44).

	Parameter	Age	PSA	Biopsy GG	PI-RADS	cT stage	*C_S_*_+_*_R_*
**Univariable**	HR	1.03	1.05	2.12	1.37	2.21	2.91
	Lower 0.95 CI	0.98	1.02	1.55	0.56	1.03	1.45
	Upper 0.95 CI	1.08	1.07	2.9	3.33	5.02	11.51
	C-index	0.53	0.69	0.72	0.64	0.67	0.78
	*p*-value	0.3	**0.03**	**0.01**	0.16	**0.02**	**0.02**
**Multivariable**	HR	0.92	1.01	1.21	1.16	2.17	4.51
	Lower 0.95 CI	0.69	0.78	0.34	0.42	1.12	1.87
	Upper 0.95 CI	1.31	1.17	2.65	3.75	4.32	14.55
	C-index		0.85 (95% CI 0.80–0.90)				
	*p*-value	0.18	**0.07**	**<0.01**	**0.06**	**0.01**	**<0.01**

HR, hazard ratio; PSA, prostate-specific antigen; GG, Gleason grade; CI, confidence interval; PI-RADS, Prostate Imaging Reporting and Data System v2.1. The values in bold indicate statistical significance.

On univariable comparison with extant assays for predicting bFS, *C_S_*_+_*_R_* resulted in higher HR (2.11, 95% CI 0.35–11.33, *p* = 0.03) compared to pre-treatment CAPRA (1.8, 95% CI 1.1–3.56, *p* = 0.02) and post-RP Decipher risk (1.41, 95% CI 0.61–2.45, *p* = 0.18); however, post-RP CAPRA-S resulted in comparable HR (2.12, 95% CI 1.2–5.72, *p* = 0.03). Integrating preoperative clinical variables with *C_S_*_+_*_R_* resulted in improved (*p* < 0.05) C-index (0.82, 95% CI 0.70–0.90) compared to CAPRA-S (0.75, 95% CI 0.69–0.78).

**Figure 5:**
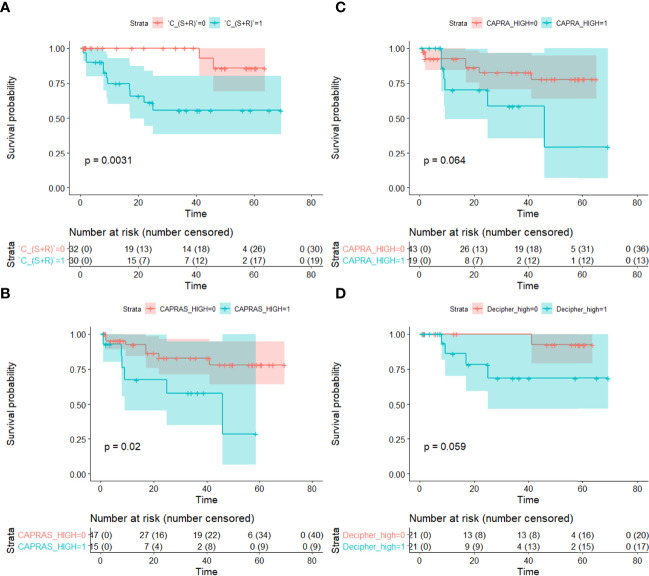
Kaplan–Meier survival curves showing differences in biochemical recurrence-free survival between BCR+ and BCR− patients based on predictions from **(A)** the integrated texture and shape classifier (*C_S_*_+_*_R_*), **(B)** CAPRA-S, **(C)** CAPRA and **(D)** Decipher risk. Statistically significant separation in BCR-free survival was observed with *C_S_*_+_*_R_* and CAPRA-S (*p* < 0.05). Statistical significance (*p* < 0.05) was established using the log-rank test.

The C-indices also follow the same trend with *C_S_*_+_*_R_* and CAPRA-S showing comparable results, and CAPRA and Decipher risk have lower values (**Table 6**). *C_S_*_+_*_R_* and CAPRA-S predictions resulted in significant separation in bFS (*p* < 0.05), while CAPRA and Decipher risk showed no significant separation on D_2_ (**Figure 5**).

**Table 6:** Comparison of the integrated radiomic model *C_S_*_+_*_R_* with post-surgical variables, nomograms CAPRA and CAPRA-S, and Decipher risk scores on the validation set (*N* = 62, *N*_BCR+_ = 13, *N*_BCR−_ = 49).

Parameter	HR	Lower 0.95 CI	Upper 0.95 CI	C-index	*p-*value
*C_S_*_+_*_R_*	2.1	0.35	11.33	0.76	**0.03**
Pathologic GG	2.6	1.16	5.93	0.82	**0.01**
EPE	2.5	1.06	11.26	0.66	**0.04**
SVI	0.8	0.24	2.57	0.49	0.7
PSM	2.9	1.32	30.2	0.71	**0.03**
CAPRA	1.8	1.1	3.56	0.69	**0.02**
CAPRA-S	2.1	1.2	5.72	0.75	**0.03**
Decipher	1.4	0.61	2.45	0.59	0.18

GG, Gleason grade; EPE, extraprostatic extension; SVI, seminal vesicle invasion; PSM, positive surgical margins; CAPRA, Cancer of the Prostate Risk Assessment (UCSF nomogram); CAPRA-S, post-surgical CAPRA. The p-values in bold indicate statistical significance.

## 4 Discussion

In this study, we presented prostate shape distension descriptors derived from a 3D shape atlas of the prostate on T2WI and explored their association with PCa BCR post-RP. We observed that the shape descriptors were prognostic of BCR and, in conjunction with radiomic texture features of PCa on T2WI and ADC maps, resulted in an improved BCR prediction model. Since shape descriptors are based on prostate segmentations, they are relatively robust to scanner variations and acquisition parameters compared to texture features. We also validated our approach on hold-out datasets that were acquired from multiple institutions validating robustness and generalizability.

Previously presented radiomic methods (17, 23, 41, 42) for predicting BCR post-RP exclusively focused on MRI texture. Our study is different from these approaches wherein we explored the shape distension of the prostate capsule within a surface of interest as a biomarker for predicting BCR. Patients experiencing BCR tend to have a relatively more aggressive phenotype of PCa (43), and hence, it appears to deform the prostate surface more substantially compared to more indolent cancers that do not result in BCR. Differential distension of the prostate capsule was observed within an SOI located toward the left posterior region, similar to the results reported by Rusu et al. (30) and Ghose et al. (31). We observed that BCR+ patients had more variations in the surface normal orientation arising out of a higher degree of prostate distension compared to BCR− patients (**Figure 3**). Prostate capsular bulge on MRI was found to be a predictor of pathologic EPE after RP by Martini et al. (44). Pathologic EPE has been shown to be a predictor of BCR in several studies (45, 46) and was observed in our study as well. Radiomic texture features including Haralick, Laws, and CoLlAGe features from T2WI and ADC sequences were the top-ranking features that were used in building *C_R_*. These features were also observed to be associated with BCR in previous works by Gnep et al. (22), Shiradkar et al. (23), and Li et al. (17).

**Figure 6:**
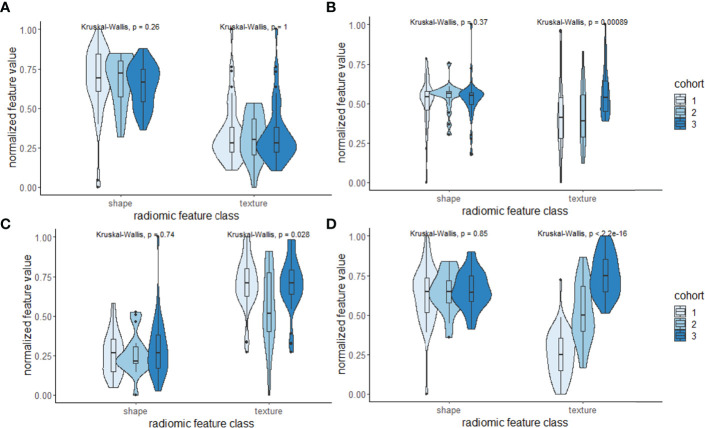
**(A–D)** Violin and box plots of the top-ranked radiomic shape and texture descriptors according to the Gini importance score within the three cohorts used in this study. Shape descriptors tend to be largely consistent and less sensitive to variations across sites compared to texture descriptors.

We observed that the shape descriptors were largely consistent between the sites (*p* > 0.05). In terms of radiomic texture descriptors, other than the Haralick feature from PCa on T2W, all the texture features had significant variations between the cohorts (*p* < 0.05) (**Figure 6**; **Table 3**). Several previous studies (26, 47, 48) exploring intersite variations in radiomic texture features have reported texture features to be sensitive to site and scanner variations. Chirra et al. (26) have shown in the context of distinguishing PCa from normal regions that Haralick features remained relatively stable while Law’s features showed significant variations which is consistent with our observations. Since radiomic shape descriptors used in our study are based on the shape of the prostate, they remain largely insensitive to site- and scanner-specific variations. This was also observed in a previous study by Merisaari et al. (49) where morphology- and shape-based radiomics were stable in terms of test–retest repeatability compared to texture features.

We observed that when compared to pre-treatment clinical variables including age, PSA, biopsy Gleason grade group (bGG), clinical T stage, or PI-RADS, *C_S_*_+_*_R_* predictions resulted in the highest HR and C-index in predicting bFS. We observed that the integrated classifier *C_S_*_+_*_R_* was independently prognostic of bFS compared to the other pre-treatment clinical variables and resulted in a high C-index in combination with those clinical variables. Post-RP Gleason grade group and EPE were however predictive of bFS on both univariable and multivariable analyses, also reported in previous studies (50, 51). The performance of the BCR predictive nomograms CAPRA and CAPRA-S in our study was in line with the results reported in previous large-scale validation studies (4). CAPRA score is derived from pre-treatment clinical variables and our radiomic shape and texture classifier *C_S_*_+_*_R_* was superior to CAPRA in predicting bFS. CAPRA-S score which includes the post-RP Gleason score resulted in a comparable performance to *C_S_*_+_*_R_* which includes pre-treatment parameters alone. On a subset of studies with the availability of Decipher in our study, we found no significant association between Decipher risk score and BCR. This was also observed in a previous study where Decipher down-classified a significant number of patients who experienced BCR (52).

We acknowledge that the study did have its limitations. Firstly, we did not explicitly control for the location of prostate cancer lesions and benign lesions and the presence of extraprostatic extension to explore their effect on prostate distension. Future studies need to be performed, involving controlling for the location of lesions in peripheral transition zones and for the size of lesions. Next, we explored inter-reader variations in prostate segmentation using only two readers and limited the SOI to the mid-gland region. Nevertheless, we observed that the shape features were robust to inter-reader and site-specific variations. We will continue to work on building a statistical model based on multireader segmentations to identify regions, where a high degree of confidence in prostate segmentation can be achieved, more precisely for subsequent shape feature analysis. We also had a smaller sample size in training and validating our approach, and our validation set had a significant imbalance between the BCR+ and BCR− classes. However, our results still generalized well over the hold-out validation set that was acquired from two different institutions. There were differences in the resolution of T2WI and ADC maps and differences in *b*-values of DWI sequences for generating ADC between the cohorts which were not explicitly accounted for in our study. We will explicitly account for the sensitivity of shape and texture radiomics to variations in resolution in our future study. We also aim to control for positive surgical margins and explore their effect on shape radiomic features in our future work.

In conclusion, radiomic shape descriptors of the prostate capsule derived from T2WI were found to be associated with BCR in our study. In combination with radiomic texture features of prostate cancer lesion from T2WI and ADC sequences, radiomic shape distension features resulted in a better predictor of BCR. Following large-scale validation studies, this approach could potentially be applied to pre-treatment prostate MRI scans at the clinic to provide clinicians with a decision support tool for assessing the risk of BCR, in turn allowing them to make better decisions for treatment management.

## Data Availability Statement

The data analyzed in this study are subject to the following licenses/restrictions: The dataset is under the IRB regulations between Case Western Reserve University, Cleveland Clinic, and the University Hospitals Cleveland Medical Center. Requests to access these datasets should be directed to rxs558@case.edu.

## Author Contributions

RS contributed to the experimental design, data acquisition, analysis, software, writing, and funding. SG provided software and contributed to the analysis. AmM helped with the data acquisition. LL provided software. IH contributed to the analysis and experiments. PF contributed to the statistical analysis. ST and AP contributed to the imaging interpretation, experimental design, and imaging analysis. LP contributed to the data acquisition, funding, and clinical interpretation. AnM contributed to the conception of the idea, experimental design, supervision, writing, and funding. All authors contributed to the article and approved the submitted version.

## Funding

The research reported in this publication was supported by the National Cancer Institute under award numbers 1U24CA199374-01, R01CA249992-01A1, R01CA202752-01A1, R01CA208236-01A1, R01CA216579-01A1, R01CA220581-01A1, R01CA257612-01A1, 1U01CA239055-01, 1U01CA248226-01, and 1U54CA254566-01; the National Heart, Lung and Blood Institute (1R01HL15127701A1); the National Institute of Biomedical Imaging and Bioengineering (1R43EB028736-01); the National Center for Research Resources under award number 1 C06 RR12463-01; VA Merit Review Award IBX004121A from the United States Department of Veterans Affairs Biomedical Laboratory Research and Development Service; the Office of the Assistant Secretary of Defense for Health Affairs, through the Breast Cancer Research Program (W81XWH-19-1-0668); the Prostate Cancer Research Program (W81XWH-15-1-0558, W81XWH-20-1-0851); the Lung Cancer Research Program (W81XWH-18-1-0440, W81XWH-20-1-0595); the Peer-Reviewed Cancer Research Program (W81XWH-18-1-0404); the Kidney Precision Medicine Project (KPMP) Glue Grant; the Ohio Third Frontier Technology Validation Fund; the Clinical and Translational Science Collaborative of Cleveland (UL1TR0002548) from the National Center for Advancing Translational Sciences (NCATS) component of the National Institutes of Health and NIH Roadmap for Medical Research; The Wallace H. Coulter Foundation Program in the Department of Biomedical Engineering at Case Western Reserve University; the DoD Prostate Cancer Research Program Idea Development Award W81XWH-18-1-0524; and the Clinical and Translational Science Collaborative (CTSC) Cleveland Annual Pilot Award 2020 UL1TR002548. This study also had sponsored research agreements from Bristol Myers-Squibb, Boehringer-Ingelheim, and AstraZeneca.

## Author Disclaimer

The content is solely the responsibility of the authors and does not necessarily represent the official views of the National Institutes of Health, the U.S. Department of Veterans Affairs, the Department of Defense, or the United States Government.

## Conflict of Interest

AnM is an equity holder in Elucid Bioimaging and Inspirata Inc. He is also a scientific advisory consultant for Inspirata Inc. In addition, he has served as a scientific advisory board member for Inspirata Inc., Astra**Z**eneca, Bristol Meyers-Squibb, and Merck. He also has sponsored research agreements with Philips and Inspirata Inc. His technology has been licensed to Elucid Bioimaging and Inspirata Inc. He is also involved in an NIH U24 grant with PathCore Inc. and three different R01 grants with Inspirata Inc.

The remaining authors declare that the research was conducted in the absence of any commercial or financial relationships that could be construed as a potential conflict of interest.

## Publisher’s Note

All claims expressed in this article are solely those of the authors and do not necessarily represent those of their affiliated organizations, or those of the publisher, the editors and the reviewers. Any product that may be evaluated in this article, or claim that may be made by its manufacturer, is not guaranteed or endorsed by the publisher.
