# Supplementary material for: Prostate Surface Distension and Tumor Texture Descriptors From Pre-Treatment MRI Are Associated With Biochemical Recurrence Following Radical Prostatectomy: Preliminary Findings
Source: Front Oncol. 2022 May 20;12:841801. doi: 10.3389/fonc.2022.841801 (PMC9163353; doi:10.3389/fonc.2022.841801)
Supplement: Supplementary file 1 [file DataSheet_1.docx]

Supplementary Material

# Supplementary Data

## Preprocessing

An N4 bias field correction (1) algorithm that estimates and subtracts the bias field was applied to T2WI acquired using endorectal coils (ERC). T2WI intensities were corrected using a previously presented standardization method aligning the histogram distributions(2). The ADC maps were co-registered to T2WI with an affine transformation using the software Elastix(3). PCa ROIs were delineated on T2WI and mapped on to ADC using the learnt transformation. In case of misaligned ADCs even after registration, a separate ROI was delineated on ADC by radiologist.

## Shape Analysis

### *Creation of BCR+ and BCR– atlases*

To create atlases for BCR+ and BCR– cohorts, all prostates inside a given subpopulation (i.e. BCR+ or BCR–) were co-registered to a representative template. This template was computed as a median volume of randomly selected 5 patient studies from each of the cohorts to minimize dependence on choice of the template. Radiologist segmented prostate ROI was used to provide anatomical constraint and improve registration accuracy. Registration of each prostate within a cohort to the representative template was performed in two stages – an initial affine registration followed by a B-spline deformable registration (4). The deformable registration was limited to 100 iterations without using a pyramidal approach and also regularized with a penalty term of 0.05 (log of the Jacobian determinant) so as to not introduce unnatural deformations in the prostate. Binary prostate ROIs were deformed following the same sequence of transformations obtained from the registered images. All co-registrations were performed using the software Elastix(3).

The registration accuracy was evaluated in terms of Dice similarity coefficient (DSC), mean absolute surface distance (MASD). DSC measures the overlap accuracy by measuring the intersection of the registered surface and penalizing for unwanted unions. MASD measures the average of absolute surface distance of the two registered surfaces. DSC is within the range [0,1] and equals unity for perfect registration. A low MASD signifies a good registration accuracy. A DSC ≥ 0.85 and MASD ≤ 0.5 mm. was employed to ascertain that the prostate was sufficiently registered. In cases with poor co-registration, additional iterations (up to 150) were employed, and the study was discarded if no significant improvement was observed.

### *Computation of surface of interest (SOI)*

To perform a statistical comparison of the prostate capsule shape between BCR+ and BCR– cohorts, all prostates were co-registered to the BCR– atlas to bring them in a common frame of reference. All registered prostate capsules of both the BCR+ and BCR– groups were isotropically resampled to 1 × 1 × 1 mm^3^ resolution and transformed into a signed distance function (SDF). The SDF gives an implicit representation of the prostate boundary and aids in a t-test based comparison of the shape in a non-parametric General Linear Model (GLM) based framework (5). A non-parametric GLM model was selected as no assumption was made about the nature of the data (mean, variance). The model parameters were learnt from the data distribution. Statistically significant shape differences were quantified with 5000 random permutation testing with the p-value being corrected for multiple comparisons. A voxel was considered as belonging to a region exhibiting statistically significant differences between shapes for BCR+ and BCR– patients if the p-value estimated by this extensive testing was less than 0.05. Significant shape differences between BCR+ and BCR– cohorts were then quantified as the surface of interest (SOI) obtained by binarizing the t-test volume at a threshold of 0.05. Co-registration was performed using the NiftyReg library (6,7) and statistical analysis to compute SOI was performed using the FSL library (8).

## Texture feature analysis

### *Details on radiomic texture feature analysis*

The T2WI and ADC maps were resampled to a 0.5 × 0.5 mm spatial resolution. A set of 75 radiomic features were extracted on a per-voxel basis from each of the T2WI and ADC maps using an in-house software (Matlab v2018b, Mathworks). These features characterize the underlying tissue heterogeneity and have previously been shown to be prognostic of BCR(9–12). Four statistics including mean, standard deviation, skewness, and kurtosis were calculated for each voxel-wise radiomic feature within each PCa ROI resulting in 75 × 2 × 4 = 600 radiomic texture descriptors per patient.

### *Feature pruning and selection*

Highly correlated radiomic features (Pearson’s correlation coefficient (R) > 0.9) were eliminated. Feature stability was computed using the metrics (13) using test-retest scans from the QIN(14) and unstable features were eliminated. This dataset contained test-retest 3T mpMRI scans of 15 PCa patients obtained over a period of 2 weeks using the same scanner. The rationale behind using these QIN cases was that stable radiomic features should remain relatively consistent across the test-retest scans of the same patient. For radiomic texture analysis, features of PCa lesions annotated separately on each scan were extracted from T2WI and ADC maps. A single reader delineated prostate ROIs on T2W MRI both on the test and re-test scans. These segmentations were then processed to obtain 3D meshes on to which the SOI was projected. Shape features within the SOI region (cropped to the mid-gland) were computed and were analyzed for differences between the test and retest scans.

We only included stable radiomic features which are identified as those without significant difference (*p* <0.05, Wilcoxon signed-rank test) between the test-retest scans. We observed no significant differences in the shape features between the test and retest scans. This suggests that the shape features may be robust to minor variations in 3D orientation of the prostate.

## Machine Learning

To account for the imbalance in classes in patients from D_1_, features from the minority class (BCR+) were oversampled using the SMOTE analysis(15). Gini importance-based feature selection was used to identify the top features associated with BCR. A 3-fold cross validation was repeated over 500 runs to determine the best classifier discriminating BCR+ and BCR– patients based on highest AUC and least standard deviation within a run. Minimum Redundancy Maximum Relevance (mRMR) Gini importance-based feature selection with random forests classifier was employed to train a radiomics texture model for BCR prediction on D_1_.

## Results

### *Evaluation of SOI where differential distension in prostate shape between BCR+ and BCR- patients is observed*

The mean DSC and MASD of co-registered BCR+ prostates across all 27 representative median templates were 0.89±0.13, 0.30±0.11 mm. and that of BCR- was 0.90±0.07, 0.40±0.14 mm. respectively, indicating a high degree of overlap suitable for statistical shape comparison. The prostate ROI on T2WI were delineated by two radiologists on a subset of 50 patient studies from D_1_. The mean DSC between prostate ROIs from two radiologists on this subset was 0.88±0.05 indicating a good agreement. The DSC was higher (0.92±0.02) when evaluated in the slices from mid-gland and was slightly lower in slices near apex (0.84±0.05) and lowest near the base (0.71±0.15) (**Supplementary Figure 1, Table 1**).

Statistically significant differences in prostate capsule shapes were observed between BCR– and BCR+ cohorts, generally towards the left posterior in the mid gland region extending towards the apex. The consensus SOI_C_ computed by averaging the binary SOIs from all templates together showed that the left posterior side in the mid gland region closer to apex most frequently appeared among all individual SOIs. The surface area of the SOI with a single template was relatively higher (2102±800 area units), but with increasing number of templates, the area of overlapped or consensus SOI falls and then gradually rises when all the templates are used to build a consensus SOI (**Figure 3**).

## Discussion

### ***Effect of inter-reader variation in prostate segmentation on shape features***

Inter-reader variation in prostate segmentation on MRI was higher in apex (dice similarity coefficient (DSC): 0.71±0.15) and base (0.84±0.05) compared to the mid gland (0.92±0.02). This can be attributed to diffuse and unclear prostate boundaries at apex and base as noted in previous studies(16,17). In order to minimize the effect of these variations, we split the prostate volume into apex (lower 25% slices), base (upper 25% slices) and the rest as mid gland. We chose this split following the approach of previous studies evaluating inter-reader variations in prostate segmentation (17,18). The SOI mapped on to individual prostates for feature extraction was cropped so that shape features from SOI were extracted only from the mid-gland region (Figure R1) which were then used to train machine learning models to predict biochemical recurrence (BCR) at 3 years. The classification performance of shape features from mid-gland region improved compared to features from the entire SOI (**Supplementary Figure 2**). The improvement was statistically significant (*p*<0.05) only in *D_1_* and not in *D­_2_*.

### *Effect of endo-rectal coil deformation on construction of atlases*

Our training set D_1_ consisted of patients scanned using an endo-rectal coil (ERC) and surface coil. The prostate meshes were normalized and resampled to the templates and any effect of ERC would be eliminated in this process. However, to control for the effect of ERC, we constructed a SOI on a subset of patients with and without an ERC and observed that the SOI was again located in the mid gland region on the left extending towards the apex as observed when all studies were considered together (**Supplementary Figure 3**).

Our training set was derived from a well curated cohort of patients such that patients in the BCR+ cohort had highly aggressive disease and presence of pathologic EPE was highly correlated to BCR outcome. This precluded us from controlling for EPE in our study, which will be a part of future work.

In patients with registration artifacts between T2WI and ADC, predictions from *C_R_* were not confident while those from *C_S_* were more confident since they did not depend on ADC signal intensity or co-registration. In patients with larger lesions and distinct hypo-intense signal intensities, the predictions from *C_R_* were more confident compared to *C_S_* (**Supplementary Figure 4**).

# Supplementary Figures and Tables

## Supplementary Figures

**Supplementary Figure 1**: Prostate segmentations were consistent between radiologists in the mid-gland region. The discrepancies were apparent at the base and apex.


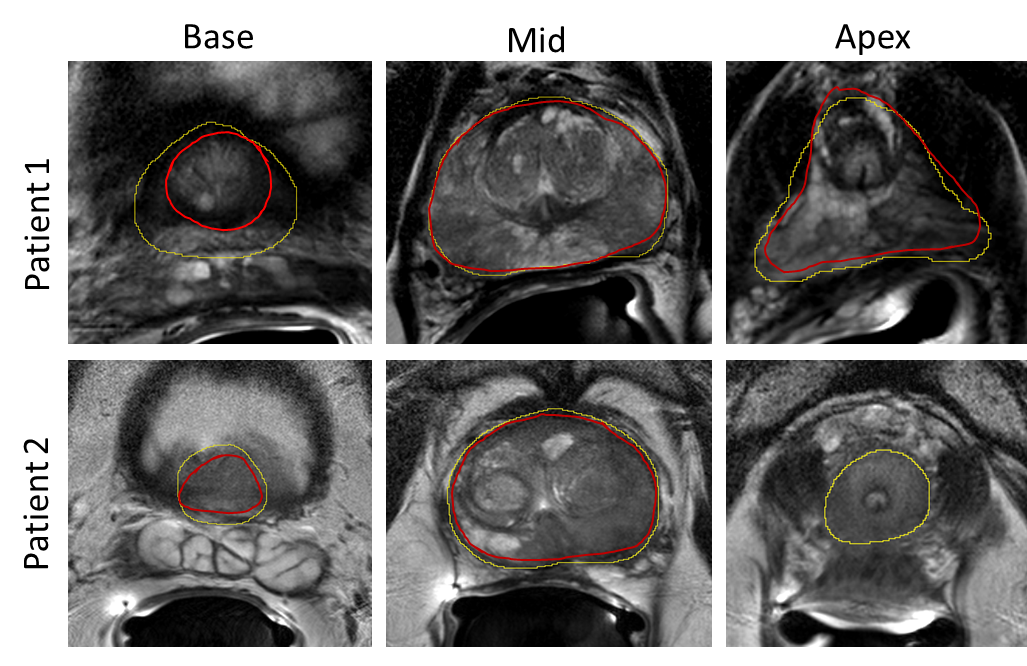

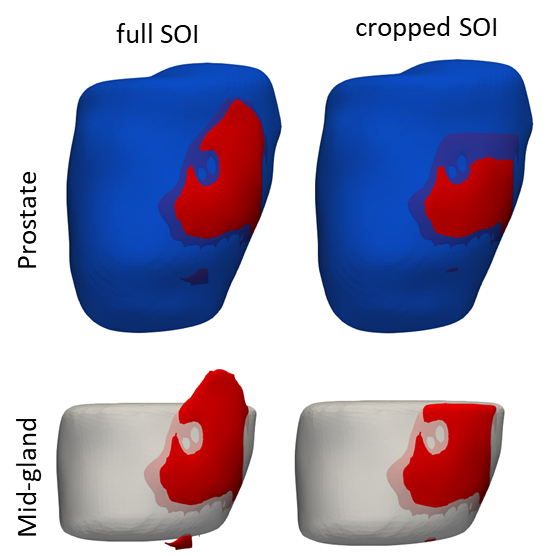


**Supplementary Figure 2a**: The SOI spans into the apex and base regions of the prostate. The cropped SOI is limited to the mid-gland region alone.


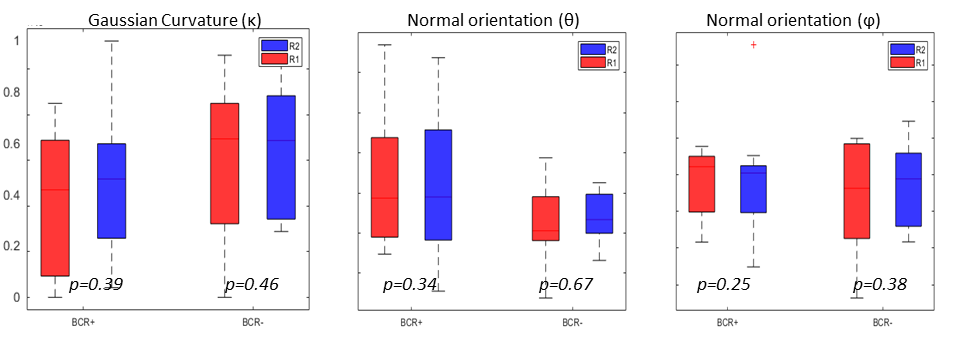


**Supplementary Figure 2b**: Inter-reader variations in shape features computed from prostate meshes derived from segmentations of two readers R1 and R2 on the validation cohort *D_2_*. We observe no significant differences in feature values within each class (BCR+/BCR-).

**Supplementary Figure 4**: Top row: Patients (P_A_ and P_B_) with registration artifacts and shape classifier *(C_S_)* was more confident of predictions compared to radiomic texture classifier *(C_R_).* Bottom row: Patients (P_C_ and P_D_) had well defined tumors and *C_R_* was confident compared to *C_S_*_._


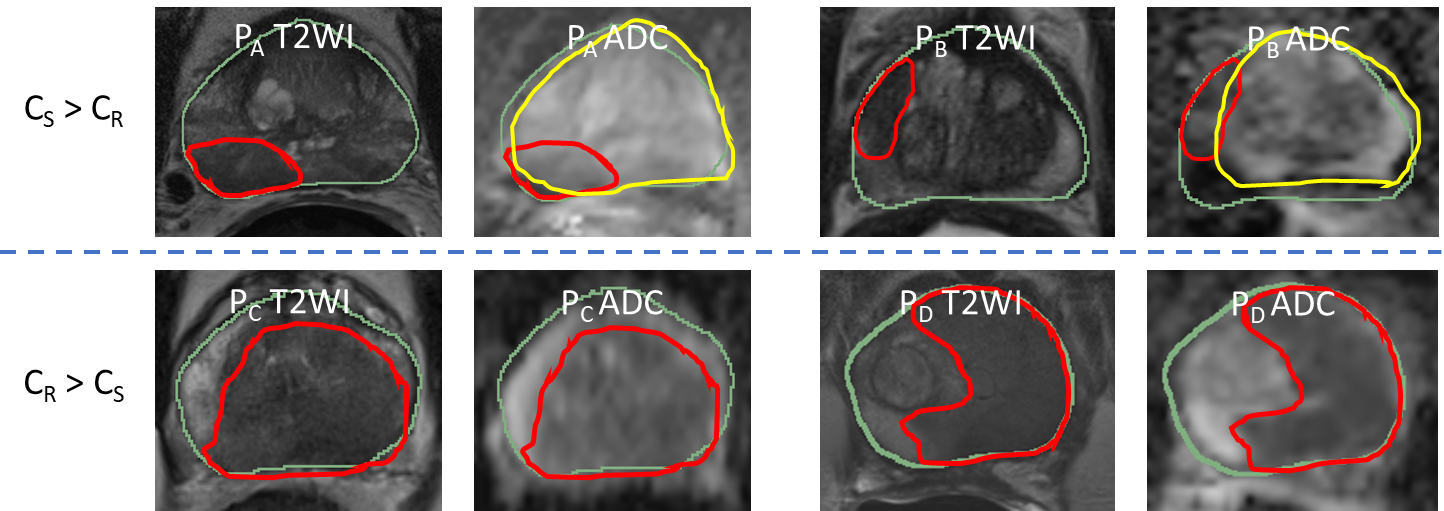

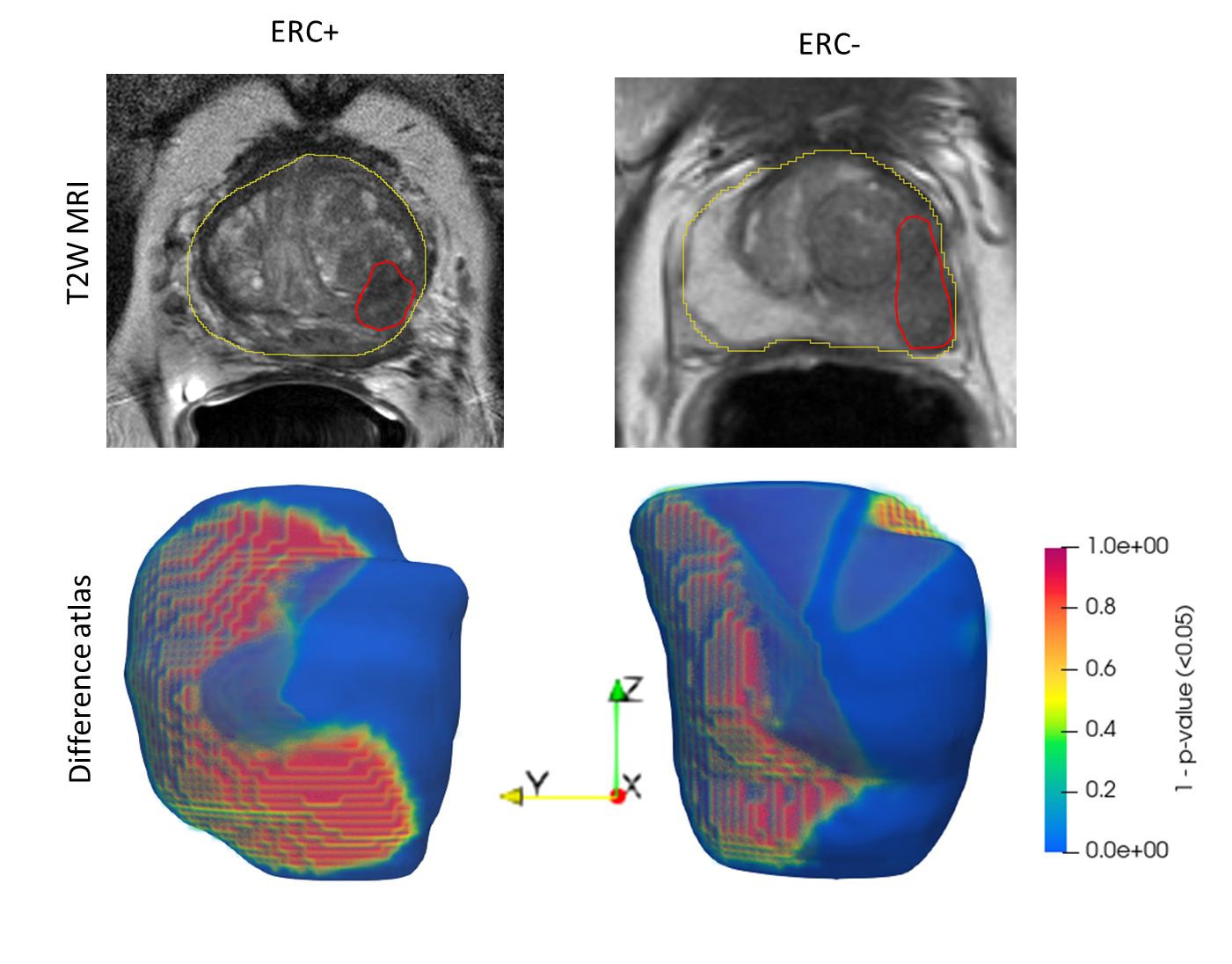


**Supplementary Figure 3:** Difference atlases computed from a sub-cohort of patients (N=14, 7 BCR+ and 7 BCR- from each of ERC+ (training) and ERC-(validation)) with and without endorectal coil (ERC). Statistically significant regions of interest are similar despite the difference in the use of ERC

## Supplementary Tables

| Supplementary Table 1: Overlap between prostate segmentation of 2 radiologists (N=50) | | |
| --- | --- | --- |
| **Zone** | **Mean DSC** | **Std DSC** |
| Base | 0.71 | 0.15 |
| Mid | 0.92 | 0.02 |
| Apex | 0.88 | 0.04 |
| Overall | 0.82 | 0.05 |
| *Note: DSC-dice similarity coefficient; std- standard deviation* | | |

| **Supplementary Table 2: Classification performance differentiating BCR+ and BCR- patients using shape feature derived from the cropped SOI and the full SOI.** | | | | |
| --- | --- | --- | --- | --- |
| **cohort** | **N_BCR+_/N_BCR-_** | **entire SOI** | **mid gland SOI** | ***p-*value (DeLong’s test)** |
|  |  | **AUC (std.)** | **AUC (std.)** |  |
| ***D_1_*** | 27/42 | 0.75 (0.69 – 0.81) | 0.78 (0.72 – 0.89) | 0.04 |
| ***D_2_ – R1*** | 13/49 | 0.68 | 0.69 | 0.12 |
| ***D_2_ – R2*** | 13/49 | 0.69 | 0.70 | 0.18 |
| *Note: D_1_ – training, D_2_ – validation, R1 & R2 – readers 1 and 2* | | | |  |

# References

1. Tustison NJ, Avants BB, Cook PA, Zheng Y, Egan A, Yushkevich PA, et al. N4ITK: improved N3 bias correction. IEEE Trans Med Imaging. 2010 Jun;29(6):1310–20.

2. Nyúl LG, Udupa JK. On standardizing the MR image intensity scale. Magn Reson Med. 1999 Dec;42(6):1072–81.

3. Klein S, Staring M, Murphy K, Viergever MA, Pluim JPW. elastix: a toolbox for intensity-based medical image registration. IEEE Trans Med Imaging. 2010 Jan;29(1):196–205.

4. Rueckert D, Sonoda LI, Hayes C, Hill DLG, Leach MO, Hawkes DJ. Nonrigid registration using free-form deformations: application to breast MR images. IEEE Transactions on Medical Imaging. 1999 Aug;18(8):712–21.

5. Winkler AM, Ridgway GR, Webster MA, Smith SM, Nichols TE. Permutation inference for the general linear model. Neuroimage. 2014 May 15;92:381–97.

6. Modat M, Cash DM, Daga P, Winston GP, Duncan JS, Ourselin S. Global image registration using a symmetric block-matching approach. J Med Imaging (Bellingham). 2014 Jul;1(2):024003.

7. Modat M, Ridgway GR, Taylor ZA, Lehmann M, Barnes J, Hawkes DJ, et al. Fast free-form deformation using graphics processing units. Comput Methods Programs Biomed. 2010 Jun;98(3):278–84.

8. Jenkinson M, Beckmann CF, Behrens TEJ, Woolrich MW, Smith SM. FSL. Neuroimage. 2012 Aug 15;62(2):782–90.

9. Gnep K, Fargeas A, Gutiérrez-Carvajal RE, Commandeur F, Mathieu R, Ospina JD, et al. Haralick textural features on T2 -weighted MRI are associated with biochemical recurrence following radiotherapy for peripheral zone prostate cancer. J Magn Reson Imaging. 2016 Jun 27;

10. Ginsburg SB, Rusu M, Kurhanewicz J, Madabhushi A. Computer extracted texture features on T2w MRI to predict biochemical recurrence following radiation therapy for prostate cancer. In: Aylward S, Hadjiiski LM, editors. 2014 [cited 2016 Oct 17]. p. 903509. Available from: http://proceedings.spiedigitallibrary.org/proceeding.aspx?doi=10.1117/12.2043937

11. Li L, Shiradkar R, Leo P, Algohary A, Fu P, Tirumani SH, et al. A novel imaging based Nomogram for predicting post-surgical biochemical recurrence and adverse pathology of prostate cancer from pre-operative bi-parametric MRI. EBioMedicine. 2021 Jan;63:103163.

12. Shiradkar R, Ghose S, Jambor I, Taimen P, Ettala O, Purysko AS, et al. Radiomic features from pretreatment biparametric MRI predict prostate cancer biochemical recurrence: Preliminary findings. J Magn Reson Imaging. 2018 May 7;

13. Khorrami M, Bera K, Leo P, Vaidya P, Patil P, Thawani R, et al. Stable and discriminating radiomic predictor of recurrence in early stage non-small cell lung cancer: Multi-site study. Lung Cancer. 2020 Apr;142:90–7.

14. Balagurunathan Y, Kumar V, Gu Y, Kim J, Wang H, Liu Y, et al. Test–Retest Reproducibility Analysis of Lung CT Image Features. J Digit Imaging. 2014 Dec;27(6):805–23.

15. Blagus R, Lusa L. SMOTE for high-dimensional class-imbalanced data. BMC Bioinformatics. 2013 Mar 22;14(1):106.

16. Becker K, Mueller JD, Schulmacher C, Ott K, Fink U, Busch R, et al. Histomorphology and grading of regression in gastric carcinoma treated with neoadjuvant chemotherapy. Cancer. 2003 Oct 1;98(7):1521–30.

17. Aldoj N, Biavati F, Michallek F, Stober S, Dewey M. Automatic prostate and prostate zones segmentation of magnetic resonance images using DenseNet-like U-net. Sci Rep. 2020 Aug 31;10(1):14315.

18. Sarma KV, Raman AG, Dhinagar NJ, Priester AM, Harmon S, Sanford T, et al. Harnessing clinical annotations to improve deep learning performance in prostate segmentation. PLOS ONE. 2021 Jun 25;16(6):e0253829.
